# From reading to writing genomes: a new direction in ELSI

**DOI:** 10.3389/fgene.2025.1667769

**Published:** 2025-09-03

**Authors:** Koichi Mikami

**Affiliations:** Department of Liberal Arts and Foreign Languages, Faculty of Science and Technology, Keio University, Yokohama, Japan

**Keywords:** genome synthesis, ELSI, re-design, collaboration, localized negotiation, proactive engagement

## Abstract

In this commentary piece, I discuss what the growing interest in synthesizing DNA at large scale means to the effort to address the Ethical, Legal and Social implications (ELSI) of genetic/genomic research. The idea that the latest scientific research should be accompanied by efforts to explore and then address its ELSI first materialized in the context of the Human Genome Project (HGP). This project to read a human genome was completed in 2003, but the science of genomics has advanced since. Particularly important was successful synthesis of *phiX174* bacteriophage genome in the very year that the HGP was concluded. This work opened up a new direction in genomics research centering on genome-scale synthesis and re-designing of genomes, characterized as a ‘writing’ approach. While early targets in this line of research were microorganisms like *Escherichia coli* and *Saccharomyces cerevisiae*, technological advancements in the synthesis of large-scale DNA sequences as well as methods to assemble them into a single genome or a chromosome are being made, and in 2016 a team of scientists proposed to ‘write’ an entire human genome. This line of scientific research, I argue, has two major characteristics, its scale and emphasis on design, and demands discussions around ‘ELSI of re-designing,’ in contrast to ELSI discussions that predominated in the earlier ‘reading’ paradigm of genomics. Because of these differences, efforts to address this ELSI of re-design should entail re-thinking what we do as ELSI as well as how we do it.

## 1 Introduction

ELSI is more than a mere abbreviation of Ethical, Legal and Social Implications of science and technology, and has become a socio-political concept with its own history. Historians have documented how James Watson, the first director of the US Human Genome Project (HGP), was prompted by concerns about social desirability of the project to introduce ELSI as a research program on the HGP’s non-scientific dimensions (e.g., Burris et al., 1998; Cook-Deegan, 1995; Juengst, 1994; Juengst, 1996; see also Watson, 1990). While the program has been quite frequently criticized (e.g., Hanna, 1995; McCain, 2002), when the HGP came to its official conclusion in 2003, Francis Collins, who succeeded Watson as director, and his colleagues at the US National Human Genome Research Institute (Collins et al., 2003) stated that ELSI research was critical to the future of genomics. And, like it or not, the program has become a template for funders to embed ethics into scientific research in the US (Fisher, 2005; 2019; Rabinow and Bennett, 2012) and abroad (Hilgartner et al., 2016; Stilgoe and Guston, 2016; Smith et al., 2023).

More than two decades have passed since then, during which the science of genomics has made considerable progress. An important milestone is the ‘more complete’ completion of human genome sequencing in 2022 (Nurk et al., 2022). Yet, more significant from an ELSI perspective may be developments in synthesizing artificial chromosomes and genomes over the last two decades (e.g., Gibson et al., 2010; Hutchson et al., 2016; Richardson et al., 2017). Although such synthesized chromosomes and genomes are primarily of microorganisms, just as the HGP started with sequencing of non-human genomes and moved on to that of human, constructing an entire human genome has even been entertained (Boeke et al., 2016). This expansion of the scope of genomics from sequencing to construction—characterized by some as a ‘writing’ approach to contrast with ‘reading’—necessarily demands re-thinking not only what should be studied under ELSI but how it should be done. This article explores how the ongoing expansion of genomics invites new directions in ELSI.

## 2 ELSI of reading the human genome

To understand the direction that ELSI research is taking, we must remember where it started. Watson introduced ELSI as a research program within the HGP, partly sensing that (bio)ethics discussions in the past might not be relevant (Fortun, 2005), but still without clear ideas about what it should study (Cook-Deegan, 1995). Policy reports from the US National Research Council and the US Office of Technology Assessment, on the feasibility and social and scientific desirability of human genome sequencing, strongly influenced Watson’s decision and suggested some areas that the ELSI program should explore, making it a kind of a policy experiment (Burris et al., 1998; Juengst, 1994; 1996).

The major concerns of the two reports related to the ambivalence expressed in the project’s original vision (US NRC Committee on Mapping and Sequencing the Human Genome, 1988; US Office of Technology Assessment, 1988). A reference genome was expected to provide a better understanding of genetic diseases, and to enable changes to be recognized that were caused by exposure to hazardous chemicals and nuclear radiation. These expectations explain why the US National Institutes of Health and US Department of Energy championed the HGP in the US. However, having a reference establishes a norm and simultaneously marks deviations from it (*cf.* Canguilhem, 1989 [1978]). Categorizing individuals with certain genetic compositions as having an inherent medical problem could be seen as reviving eugenics, which the genetic research community had tried to dissociate themselves from since the end of World War II (Keller, 1992; Kevles, 1995). Hence, a danger was intrinsic to the core vision of the HGP.

ELSI, as a way to manage this danger, was often discussed with more sophistication than bluntly questioning whether human genome sequencing would revive eugenics. This was partly due to the effort of the medical genetics community in the 1970s to develop genetic counseling practices that respect patient and family autonomy (see Stern, 2012). At the same time, it remained true for most genetic diseases—excepting a small number that can be managed by life-long dietary control and palliative treatment—that identifying them through prenatal testing was only useful in making decisions about birth control and abortion. Although the emphasis was on enabling individual families to make the best decision for them, not eradicating ‘bad’ genes from the human population, genetic counseling was still about the possibility of eliminating them in one’s family. This kind of discussion can be viewed as ‘ELSI of eradication,’ which remains an important component of ELSI of genomics.

A second kind of ELSI of genomics can be framed, in contrast, as ‘ELSI of knowing,’ including, for example, the issue of incidental findings in research. By emphasizing patient autonomy, medical genetics tended to assume that knowledge equaled empowerment to make an informed decision. This assumption was challenged when genetic testing for Huntington disease was developed in the mid-1980s. Scientists suggested that patients might seriously suffer from knowing that they had inherited the gene for the disease, as no effective treatment was available (Hotlzman, 1989). Concern also arose about what this knowledge might mean for job markets and insurance schemes. Genome sequencing, together with improvements in early genetic testing, have thus raised critical questions about who should have such knowledge and how it should be used. The US Genetic Information Non-discrimination Act of 2008 can be seen as a preeminent example of a social intervention resulting from this kind of ELSI discussion.

As Juengst explains, ELSI discussions in this era aimed “to develop well-informed professional and public policy options on the issues in its domain and to convey these recommendations to the public in an effective fashion” (1994, p.122). ELSI discussions under the ‘reading’ approach to human genomics also addressed other questions, but these two areas have been particularly significant. Both ELSI of eradication and of knowing centers concerns about the ambivalence of having knowledge about the presence (or absence) of a certain gene or set of genes versus having the ability to act on that knowledge. They indicate that ELSI emerged as human-centric discussions in what we might characterize as a knowledge-based anticipation and control paradigm (Juengst, 2021). The ELSI research program of the HGP therefore prompted social scientists and humanities scholars to address possibilities and dangers associated with this newly increased capacity to know about our genome and act on the knowledge.

## 3 The science of writing a genome

A ‘writing’ approach in genomics emerged at around the time the HGP was coming to an end. The history of DNA synthesis goes back to the 1950s (Michaelson and Todd, 1955; Gilham and Khorana, 1958; see also Berry, 2019), making it almost as old as the discovery of double helix structure of DNA. Then in the late 1970s, around the same time as the development of Sanger sequencing method, synthesis of a gene was first achieved, but it was only 207 base-pairs long (Khorana, 1979). In 2003, the very same year as the HGP was concluded, the new approach took off as a group led by J. Craig Venter announced that it had successfully synthesized the genome of *phiX174* bacteriophage, approximately 5,000 base-pairs long (Smith et al., 2003). With a prospect of assembling synthetic DNA segments of similar length into an even larger microbial genome, the group’s success opened up the possibility of genome-scale synthesis.

The group formed the J. Craig Venter Institute (JCVI) in 2006, and went on to synthesize the genome of a bacterium *Mycoplasma genitalium* in 2008 (Gibson et al., 2008). Though the smallest-known genome of any self-replicating organism, at 582,970 base-pairs, it is still 100-fold larger than *phiX174* bacteriophage. Then only a couple of years later, the JCVI constructed the even larger 1.08 megabase-pair *Mycoplasma myocides* genome (Gibson et al., 2010). Furthermore, the group transplanted this synthesized genome into a different sub-species and created a ‘new’ organism *M. mycoides JCVI syn1.0*, demonstrating that a synthetic genome could sustain a living cell in the same way as its natural template does.

Scale is a key feature of this new ‘writing’ approach in genomics, and another important characteristic is its emphasis on design. The process of genome synthesis involved designing DNA cassettes for assembly as well as insertion of short ‘watermark’ sequences to differentiate the synthetic genome from the original. Design considerations became much more prominent in *JCVI syn3.0*, released in 2016 (Hutchson et al., 2016)—a re-designed version of *M. mycoides JCVI syn1.0* with a ‘minimal genome’ containing only the ‘essential’ 473 genes for supporting life (under controlled laboratory conditions).

The ‘writing’ approach in genomics expanded in the 2010s. In 2016, a genomically recoded *Escherichia coli* which uses only 57 of the canonical 64 codons was proposed (Ostrov et al., 2016). The design concept was based on earlier research to replace all TAG stop codons across the bacterial genome with synonymous TAA codons (Isaac et al., 2010), assigning TAG codons a new nonstandard amino acid (Lajoie et al., 2013). In 2019, a group in the UK created a synthetic *E. coli* strain, called *Syn61*, which uses only 61 codons to encode the canonical 20 amino acids (Fredens et al., 2019). The TAG-TAA codon replacement was also deployed, along with another design principle called SCRaMbLE to generate large-scale genome-rearrangements, in Sc2.0, a project to synthesize all the 16 chromosomes of *Saccharomyces cerevisiae* (Dymond et al., 2011; Annaluru et al., 2014; Richardson et al., 2017).

From the very beginning of the ‘writing’ approach, its core vision was and has been to address important biological questions that would not be answered by other approaches including that of ‘reading’ (Smith et al., 2003). The vision explains why microorganisms like *E. coli* and *S. cerevisiae* have been popular targets of the ‘writing’ approach. Just as sequencing of their genome in the ‘reading’ approach helped them to consolidate their status as model organisms (Leonelli and Ankeny, 2013), these microorganisms are chosen to be useful platforms for future genomics research (e.g., Yoneji et al., 2021). Therefore, the design concepts applied to them are explained as ways to give resultant new organisms “genetic flexibility to facilitate future studies” (Dymond et al., 2011, p.471). It has been suggested that the ‘writing’ approach has presented scientists with “a powerful new ‘hammer’” to crack the mechanism of “how life works” (Endy, 2008, p.1197).

## 4 Discussion

That the scope of genomics is expanding fast does not mean that ELSI discussions of the ‘reading’ approach is outmoded. Its importance has even increased by advance in genetic engineering technologies, and particularly by the development of CRISPR-Cas9 (Jinek et al., 2012). This technology functions like genetic scissors to cut out targeted DNA sequence in a genome easily and precisely (Doudna and Charpentier, 2014). If it becomes available for therapeutic use, the knowledge of having a genetic disease, including Huntington disease, will have a different implication (Baylis, 2019). More concerning is its use on human germline cells, not only because changes made to the genome will be passed down to future generations but also because some such changes may be intended for enhancement (National Academy of Sciences, 2017; National Academy of Sciences, 2020). The progress in this area of genomics is transforming the ability to act on the knowledge of genetic compositions. Yet, as long as its focus is on insertion and/or deletion of functional genes, the technology remains in a knowledge-based anticipation and control paradigm, and ELSI of eradication and of knowing continues to be highly relevant for its governance.

In contrast, attempts to use the same technology for genome-scale manipulation (e.g., Smith et al., 2020; Liu et al., 2022; Koeppel et al., 2025) add urgency to ELSI discussions of the ‘writing’ approach. Although microorganisms have been its early targets, the idea of launching an international consortium like the HGP to synthesize human genome has been entertained (Boeke et al., 2016). Given the sizes of synthetic genomes produced so far, the goal may appear unattainable in the foreseeable future, but its proponents suggested that technical advancement and cost reduction are more likely to be achieved by working toward the big challenge together, as happened in the HGP. And attempts have already been made to introduce design principles like SCRaMbLE to human cell lines, not by synthesizing the genome but by genome-scale editing (Koeppel et al., 2025). Also, as growing interest in the human microbiome after the HGP (e.g., The NIH HMP Working Group, 2009) indicates, what make us human cannot be reduced to the human genome alone. Linking to conversations around multispecies ethnography, which emphasizes situatedness of our being in the network of many other living and non-living entities, in social sciences, Szymanski (2023) urges us to think that various efforts to create synthetic non-human organisms are leading to a path to ‘indirect’ human engineering.

What then should ELSI of this ‘writing’ approach look like? Informed by its two key characteristics, namely, its scale and emphasis on design, it may be productive to frame the kind of ELSI discussions it requires as ‘ELSI of re-designing.’ The point is that new organisms, human or not, with significantly altered biological systems are being created, and the process of their creation necessarily involves decisions about their design. The decision often takes a form of a series of choices, and these choices, even if they appear to be only technical, necessarily involve values, for example, about what is useful or what is interesting. Also, as indicated by the notion of ‘indirect’ human engineering, the decision may be made without knowing what implications it may have beyond the organism to be created. Therefore, there are always discussions to be had about the choices themselves as well as who has the power to make them (Calvert and Szymanski, 2020). And the aim of such discussions ought to be ensuring different values feed into in the process of arriving at the decisions, rather than merely attempting to manage its consequences primarily by developing informed policy options.

From this point of view, ELSI of re-designing resonates with the ideas of ‘anticipatory governance’ and ‘responsible research and innovation (RRI)’ emerged in the broader trend of science and technology governance since the ending of the HGP (e.g., Stilgoe and Guston, 2016; Fisher, 2019). Anticipatory governance, discussed primarily in the context of nanotechnology research, aims to shape the trajectory of the technology by developing capacities of foresight to explore future possibilities, engagement for the voices of nonexpert stakeholders to be heard and integration of collective decision-making opportunities in the process of technological development (Barben et al., 2008; Guston, 2014). Similarly, RRI aspires to increase societal desirability of research and innovation by improving capacities for anticipation, inclusion, reflexivity and responsiveness (Owen et al., 2012; Stilgoe et al., 2013; see also Boeke et al., 2016). Stressed in these ideas is the importance of achieving “a better alignment between goals of science, technology and innovation and those of diverse publics” (Ribeiro et al., 2018, p.318).

It is important to note, however, that different values to be considered in the ‘writing’ approach are not restricted to those of humans. Microorganisms like *E. coli* and *S. cerevisiae* are frequently used in this approach, because of their favorable characteristics, such as small genome sizes and short generation times (Leonelli and Ankeny, 2013). In the production of *M. genitalium* genome, for instance, *E. coli* was used to clone DNA segments in the early stages of the assembly, as the bacteria have been used for this kind of work for decades. Yet, the researchers encountered difficulties in carrying out the planned assemblies in the organism and needed to resort to *S. cerevisiae* for the final stages (Gibson et al., 2008, p.1216). In attempts to ‘write’ at the scale of genome, therefore, the organisms involved can have significant implications for what is to be created. This observation invites us to think that in the ‘writing’ approach, scientists must work *with* the organisms they hope to re-design (Calvert and Szymanski, 2020). This style of science not only demands attentiveness to biological as well as cultural idiosyncrasies of individual organisms but also calls transferability of its outcomes into question as they are local achievements attained with their contribution.

And another kind of collaborators who are present but often unrecognized as valuable contributors to the science is scholars of social sciences and humanities. As mentioned earlier, the conventional mode of ELSI research was to engage discussions on the possibilities and dangers associated with advances in genomics, *as outsiders*. Despite the vision to integrate them in scientific research, as *collaborators*, however, their role often remains to be exploring societal concerns of the knowledge and/or technology to be produced (Fisher, 2019). Provided that re-designing organisms is achieved through localized negotiation of what is to be created, they should engage in the negotiation as autonomous experts with their own set of skills and values, rather than merely trying to speak for society at large. They can also bring in more affirmative affects, such as excitement, enjoyment, surprise and interest, to ELSI discussions, rather than solely negative ones like skepticism, opposition and distrust (see Fortun, 2005). Such proactive engagement may be considered ‘complicit’ potentially causing them discomfort (see Calvert, 2013), but it is important to remember that disengagement is also a kind of engagement with influence on the decisions to be made.

The ‘writing’ approach in genomics is an exploratory endeavor situated in local contexts of the science being done. Every work is achieved through localized negotiation among human and non-human collaborators, who may not necessarily share the same goal, but still such negotiation may change who we all are. Therefore, its ELSI discussions can neither be human-centric nor happen in the knowledge-based anticipation and control paradigm, unlike those of the ‘reading’ approach. They are critical part of the negotiation. Recognizing the agency of those who were deemed invisible and voiceless previously, while accepting the limit of the values they can bring in to the process, is a modest but important step toward making different values count in advancing the science of genomics.

## Data Availability

The original contributions presented in the study are included in the article/supplementary material, further inquiries can be directed to the corresponding author.
